# Supplementary material for: Plant parentage influences the type of timber use by traditional peoples of the Brazilian Caatinga
Source: PLoS One. 2023 Oct 17;18(10):e0286434. doi: 10.1371/journal.pone.0286434 (PMC10581497; doi:10.1371/journal.pone.0286434)
Supplement: S1 Table — (DOCX) [file pone.0286434.s001.docx]

Supporting information**:** List with the name of the species and wood usage considered in the study, and the works consulted to collect data on wood density.

| **Plant species** | **Reference** |
| --- | --- |
| *Amburana cearensis* | Lorenzi, H.1992. Árvores brasileiras: manual de identificação e cultivo de plantas arbóreas nativas do Brasil. 3. ed. Nova Odessa: Plantarum. |
| *Anadenanthera colubrina* | Silva, A.M.L. (2012). Tipos funcionais de plantas no semiárido: Quais são os atributos chave?Dissertação de mestrado. Universidade Estadual da Paraíba. |
| *Annona leptopetala* | Pinho, B., Zorger, B., Rosado, B,, & Tabarelli, M. (2019). Functional organization of woody plant assemblages along precipitation and human disturbance gradients in a seasonally dry tropical forest. Dryad. Dataset, <https://doi.org/10.5061/dryad.jwstqjq4r> |
| *Aspidosperma pyrifolium* | Silva, A.M.L. (2012). Tipos funcionais de plantas no semiárido: Quais são os atributos chave?Dissertação de mestrado. Universidade Estadual da Paraíba. |
| *Astronium urundeuva* | Silva, A.M.L. (2012). Tipos funcionais de plantas no semiárido: Quais são os atributos chave?Dissertação de mestrado. Universidade Estadual da Paraíba. |
| *Bauhinia cheilantha* | Silva, A.M.L. (2012). Tipos funcionais de plantas no semiárido: Quais são os atributos chave?Dissertação de mestrado. Universidade Estadual da Paraíba. |
| *Ceiba glaziovii* | Lorenzi, H.1992. Árvores brasileiras: manual de identificação e cultivo de plantas arbóreas nativas do Brasil. 3. ed. Nova Odessa: Plantarum. |
| *Cenostigma pyramidale* | Silva, A.M.L. (2012). Tipos funcionais de plantas no semiárido: Quais são os atributos chave?Dissertação de mestrado. Universidade Estadual da Paraíba. |
| *Chloroleucon mangense* | Pinho, B., Zorger, B., Rosado, B,, & Tabarelli, M. (2019). Functional organization of woody plant assemblages along precipitation and human disturbance gradients in a seasonally dry tropical forest. Dryad. Dataset, <https://doi.org/10.5061/dryad.jwstqjq4r> |
| *Cnidoscolus quercifolius* | Lorenzi, H.1992. Árvores brasileiras: manual de identificação e cultivo de plantas arbóreas nativas do Brasil. 3. ed. Nova Odessa: Plantarum. |
| *Combretum leprosum* | Rocha, E.L.B. (2018). Indicação de madeiras da caatinga para uso na produção de brinquedo. Natural Resources, 8(1), 9-16. <http://doi.org/10.6008/CBPC2237-9290.2018.001.0002> |
| *Commiphora leptophloeos* | Lorenzi, H.1992. Árvores brasileiras: manual de identificação e cultivo de plantas arbóreas nativas do Brasil. 3. ed. Nova Odessa: Plantarum. |
| *Cordia trichotoma* | Ribeiro, E.M.S., Lohbeck, M., Santos B.A., Arroyo-Rodríguez, V., Tabarelli, M., Leal, I.A. (2019). ata from: Functional diversity and composition of Caatinga woody flora are negatively impacted by chronic anthropogenic disturbance, Dryad, Dataset, <https://doi.org/10.5061/dryad.0d1s343> |
| *Coutarea hexandra* | attge, J., Díaz, S., Lavorel, S., Prentice, I.C., Leadley, P., Bönisch, G., Garnier, E., Westoby, M., Reich, P.B., Wright, I.J., Cornelissen, J.H.C., Violle, C., Harrison, S.P., Van Bodegom, P.M., Reichstein, M., Enquist, B.J., Soudzilovskaia, N.A., Ackerly, D.D., Anand, M.,... & Wirth, C. (2011). TRY - Um banco de dados global de características de plantas. Global Change Biology*,* 17 (9), 2905-2935. <https://doi.org/10.1111/j.1365-2486.2011.02451.x> |
| *Croton blanchetianus* | Lima and Silva, A.M., Lopes, S.F., Vitorio, L.A.P., Santiago, R.R., Mattos, E.A., & Trovão, D.M.B.M. 2014. Plant functional groups of species in semiarid ecosystems in Brazil: wood basic density and SLA as an ecological indicator. Brazilian Journal of Botany, 37, 229-237. https://10.1007/s40415-014-0063-4. |
| *Croton heliotropiifolius* | Lima and Silva, A.M., Lopes, S.F., Vitorio, L.A.P., Santiago, R.R., Mattos, E.A., & Trovão, D.M.B.M. 2014. Plant functional groups of species in semiarid ecosystems in Brazil: wood basic density and SLA as an ecological indicator. Brazilian Journal of Botany, 37, 229-237. https://10.1007/s40415-014-0063-4. |
| *Cynophalla flexuosa* | Silva, A.M.L. (2012). Tipos funcionais de plantas no semiárido: Quais são os atributos chave?Dissertação de mestrado. Universidade Estadual da Paraíba. |
| *Dahlstedtia araripensis* | Trindade, D.P.F. (2017). Montagem de comunidades durante a sucessão secundária na caatinga: efeito da limitação de dispersão e recrutamento de plantas. Dissertação de mestrado. Universidade Federal de Pernambuco. Centro de Biociências. Programa de pós-graduação em Biologia Vegetal. |
| *Enterolobium timbouva* | Lorenzi, H.1992. Árvores brasileiras: manual de identificação e cultivo de plantas arbóreas nativas do Brasil. 3. ed. Nova Odessa: Plantarum. |
| *Erythrina velutina* | Butz, P., Raffelsbauer, V., Graefe, S., Peters, T., Cueva, E., Holscher, D., & Brauning, A. (2017). Tree responses to moisture fluctuations in a neotropical dry forest as potential climate change indicators, Ecological Indicators, 83. <https://doi.org/10.1016/j.ecolind.2016.11.021>. |
| *Euphorbia tirucalli** | Nascimento, I.S. (2016). A coordenação funcional entre os diferentes órgãos das plantas arbóreas da floresta atlântica varia conforme e estratégia de uso e conservação dos recursos? Dissertação. Mestrado em Ciências Florestais |
| *Guapira hirsuta** | Nascimento, I.S. (2016). A coordenação funcional entre os diferentes órgãos das plantas arbóreas da floresta atlântica varia conforme e estratégia de uso e conservação dos recursos? Dissertação. Mestrado em Ciências Florestais |
| *Guapira laxa** | Nascimento, I.S. (2016). A coordenação funcional entre os diferentes órgãos das plantas arbóreas da floresta atlântica varia conforme e estratégia de uso e conservação dos recursos? Dissertação. Mestrado em Ciências Florestais |
| *Handroanthus impetiginosus* | Pinho, B., Zorger, B., Rosado, B,, & Tabarelli, M. (2019). Functional organization of woody plant assemblages along precipitation and human disturbance gradients in a seasonally dry tropical forest. Dryad. Dataset, <https://doi.org/10.5061/dryad.jwstqjq4r> |
| *Hymenaea courbaril* | Lorenzi, H.1992. Árvores brasileiras: manual de identificação e cultivo de plantas arbóreas nativas do Brasil. 3. ed. Nova Odessa: Plantarum. |
| *Jatropha molíssima* | Lima, A.L.A. (2010). Tipos funcionais fenológicos de espécies lenhosas da Caatinga, nordeste do Brasil. Tese. Programa de Pós Graduação em Botânica. Universidade Federal Rural de Pernambuco. |
| *Libidibia ferrea* | Silva, A.M.L. (2012). Tipos funcionais de plantas no semiárido: Quais são os atributos chave?Dissertação de mestrado. Universidade Estadual da Paraíba. |
| *Luetzelburgia auriculata* | Machado-Neto, A.P., Brandão, C.F.L.S., Duarte, B., Almir, J., Marangon, L.C., & Feliciano, A.L.P. (2015). Densidade e Poder Calorífico como Base para Prevenção de Incêndios Florestais sob Linhas de Transmissão. NATIVA, 3:10-15. |
| *Manihot glaziovii** | Lima, A.L.A. (2010). Tipos funcionais fenológicos de espécies lenhosas da Caatinga, nordeste do Brasil. Tese. Programa de Pós Graduação em Botânica. Universidade Federal Rural de Pernambuco. |
| *Mimosa arenosa* | Paes, J.B., Lima, C.R., Oliveira, E., & Medeiros-Neto, P.N. (2013). Características Físico-Química, Energética e Dimensões das Fibras de Três Espécies Florestais do Semiárido Brasileiro. Floresta e Ambiente, 20(4): 550-555. |
| *Mimosa caesalpiniifolia* | Gonçalves, C.A., Lelis, R.C.C., & Abreu, H.S. (2010). Caracterização físico-química da madeira de sabiá (*Mimosa caesalpiniaefolia* Benth.). Revista Caatinga, Mossoró, 23(1), 54-62. |
| *Mimosa lewisii* | Pinho, B., Zorger, B., Rosado, B,, & Tabarelli, M. (2019). Functional organization of woody plant assemblages along precipitation and human disturbance gradients in a seasonally dry tropical forest. Dryad. Dataset, <https://doi.org/10.5061/dryad.jwstqjq4r> |
| *Mimosa ophthalmocentra* | Silva, A.M.L. (2012). Tipos funcionais de plantas no semiárido: Quais são os atributos chave?Dissertação de mestrado. Universidade Estadual da Paraíba. |
| *Mimosa tenuiflora* | Silva, A.M.L. (2012). Tipos funcionais de plantas no semiárido: Quais são os atributos chave?Dissertação de mestrado. Universidade Estadual da Paraíba. |
| *Piptadenia retusa* | Silva, A.M.L. (2012). Tipos funcionais de plantas no semiárido: Quais são os atributos chave?Dissertação de mestrado. Universidade Estadual da Paraíba. |
| *Sapium glandulosum* | Lima, A.L.A. (2010). Tipos funcionais fenológicos de espécies lenhosas da Caatinga, nordeste do Brasil. Tese. Programa de Pós Graduação em Botânica. Universidade Federal Rural de Pernambuco. |
| *Sarcomphalus joazeiro* | Ribeiro, E.M.S., Lohbeck, M., Santos B.A., Arroyo-Rodríguez, V., Tabarelli, M., Leal, I.A. (2019). ata from: Functional diversity and composition of Caatinga woody flora are negatively impacted by chronic anthropogenic disturbance, Dryad, Dataset, <https://doi.org/10.5061/dryad.0d1s343> |
| *Schinopsis brasiliensis* | Silva, A.M.L. (2012). Tipos funcionais de plantas no semiárido: Quais são os atributos chave?Dissertação de mestrado. Universidade Estadual da Paraíba. |
| *Senna spectabilis** | Ribeiro, E.M.S., Lohbeck, M., Santos B.A., Arroyo-Rodríguez, V., Tabarelli, M., Leal, I.A. (2019). ata from: Functional diversity and composition of Caatinga woody flora are negatively impacted by chronic anthropogenic disturbance, Dryad, Dataset, <https://doi.org/10.5061/dryad.0d1s343> |
| *Sideroxylon obtusifolium* | Silva, A.M.L. (2012). Tipos funcionais de plantas no semiárido: Quais são os atributos chave?Dissertação de mestrado. Universidade Estadual da Paraíba. |
| *Spondias tuberosa* | Lima, A.L.A., Sampaio, E.V.S.B., Castro, C.C., Rodal, M.J.N., Antonino, C.D., & Melo, A.L. (2012). Do the phenology and functional stem attributes of woody species allow for the identification of functional groups in the semiarid region of Brazil? Brazil123Trees, 26:1605–1616DOI 10.1007/s00468-012-0735-2 |
| *Tabebuia aurea* | Lorenzi, H.1992. Árvores brasileiras: manual de identificação e cultivo de plantas arbóreas nativas do Brasil. 3. ed. Nova Odessa: Plantarum. |
| *Ximenia americana* | Ribeiro, E.M.S., Lohbeck, M., Santos B.A., Arroyo-Rodríguez, V., Tabarelli, M., Leal, I.A. (2019). ata from: Functional diversity and composition of Caatinga woody flora are negatively impacted by chronic anthropogenic disturbance, Dryad, Dataset, <https://doi.org/10.5061/dryad.0d1s343> |
| *Zanthoxylum rhoifolium* | Silva, A.M.L. (2012). Tipos funcionais de plantas no semiárido: Quais são os atributos chave?Dissertação de mestrado. Universidade Estadual da Paraíba. |
