# Supplementary material for: Plant parentage influences the type of timber use by traditional peoples of the Brazilian Caatinga
Source: PLoS One. 2023 Oct 17;18(10):e0286434. doi: 10.1371/journal.pone.0286434 (PMC10581497; doi:10.1371/journal.pone.0286434)
Supplement: Attachment — Submitted filename: response letters.docx [file pone.0286434.s002.docx]

To:  Dr. Tzen-Yuh Chiang

Academic Editor of PLOS ONE

Thank you for potentially accepting our manuscript entitled **“Plant parentage influences the type of timber use by traditional peoples of the Brazilian Caatinga”** for publication in PLOS ONE.

We would like to acknowledge the excellent suggestions from both reviewers for improving the revised version of our manuscript.

The suggested edits and comments made by the reviewers are shown below.

Reviewers' comments:

**Journal requirements:**

When submitting your revision, we need you to address these additional requirements.

1.  Please ensure that your manuscript meets PLOS ONE's style requirements, including those for file naming. The PLOS ONE style templates can be found at

<https://journals.plos.org/plosone/s/file?id=wjVg/PLOSOne_formatting_sample_main_body.pdf>  and

<https://journals.plos.org/plosone/s/file?id=ba62/PLOSOne_formatting_sample_title_authors_affiliations.pdf>

**Response:** done.

2. Please include your amended statements within your cover letter; we will change the online submission form on your behalf.

**Response:** Thank you for the instructions. We certify that the paper is in the style of PLOS ONE. We removed the funding from the acknowledgements session. Below we describe our funding source:

Funding Statement: MBR and KMP thank the Fundação de Apoio à Pesquisa do Estado da Paraíba (FAPESq) for granting research grants. This study was partly funded by the State University of Paraíba grant 03/2022. SFL thanks CNPq for the productivity grant.

3. In your Data Availability statement, you have not specified where the minimal data set underlying the results described in your manuscript can be found. PLOS defines a study's minimal data set as the underlying data used to reach the conclusions drawn in the manuscript and any additional data required to replicate the reported study findings in their entirety. All PLOS journals require that the minimal data set be made fully available. For more information about our data policy, please see http://journals.plos.org/plosone/s/data-availability.

Upon re-submitting your revised manuscript, please upload your study’s minimal underlying data set as either Supporting Information files or to a stable, public repository and include the relevant URLs, DOIs, or accession numbers within your revised cover letter. For a list of acceptable repositories, please see http://journals.plos.org/plosone/s/data-availability#loc-recommended-repositories. Any potentially identifying patient information must be fully anonymized.

Important: If there are ethical or legal restrictions to sharing your data publicly, please explain these restrictions in detail. Please see our guidelines for more information on what we consider unacceptable restrictions to publicly sharing data: http://journals.plos.org/plosone/s/data-availability#loc-unacceptable-data-access-restrictions. Note that it is not acceptable for the authors to be the sole named individuals responsible for ensuring data access.

We will update your Data Availability statement to reflect the information you provide in your cover letter.

**Response:** Thank you for the instructions. The data generated and analyzed for the current study has been uploaded to the Dryad Digital Repository <https://doi.org/10.5061/dryad.w3r2280w5>

4. We note that Figure 1 in your submission contain [map/satellite] images which may be copyrighted. All PLOS content is published under the Creative Commons Attribution License (CC BY 4.0), which means that the manuscript, images, and Supporting Information files will be freely available online, and any third party is permitted to access, download, copy, distribute, and use these materials in any way, even commercially, with proper attribution. For these reasons, we cannot publish previously copyrighted maps or satellite images created using proprietary data, such as Google software (Google Maps, Street View, and Earth). For more information, see our copyright guidelines: http://journals.plos.org/plosone/s/licenses-and-copyright.

We require you to either (1) present written permission from the copyright holder to publish these figures specifically under the CC BY 4.0 license, or (2) remove the figures from your submission:

a. You may seek permission from the original copyright holder of Figure 1 to publish the content specifically under the CC BY 4.0 license.

We recommend that you contact the original copyright holder with the Content Permission Form (http://journals.plos.org/plosone/s/file?id=7c09/content-permission-form.pdf) and the following text:

“I request permission for the open-access journal PLOS ONE to publish XXX under the Creative Commons Attribution License (CCAL) CC BY 4.0 (http://creativecommons.org/licenses/by/4.0/). Please be aware that this license allows unrestricted use and distribution, even commercially, by third parties. Please reply and provide explicit written permission to publish XXX under a CC BY license and complete the attached form.”

Please upload the completed Content Permission Form or other proof of granted permissions as an "Other" file with your submission.

In the figure caption of the copyrighted figure, please include the following text: “Reprinted from [ref] under a CC BY license, with permission from [name of publisher], original copyright [original copyright year].”

b. If you are unable to obtain permission from the original copyright holder to publish these figures under the CC BY 4.0 license or if the copyright holder’s requirements are incompatible with the CC BY 4.0 license, please either i) remove the figure or ii) supply a replacement figure that complies with the CC BY 4.0 license. Please check copyright information on all replacement figures and update the figure caption with source information. If applicable, please specify in the figure caption text when a figure is similar but not identical to the original image and is therefore for illustrative purposes only.

The following resources for replacing copyrighted map figures may be helpful:

USGS National Map Viewer (public domain): http://viewer.nationalmap.gov/viewer/

The Gateway to Astronaut Photography of Earth (public domain): http://eol.jsc.nasa.gov/sseop/clickmap/

Maps at the CIA (public domain): https://www.cia.gov/library/publications/the-world-factbook/index.html and https://www.cia.gov/library/publications/cia-maps-publications/index.html

NASA Earth Observatory (public domain): http://earthobservatory.nasa.gov/

Landsat: http://landsat.visibleearth.nasa.gov/

USGS EROS (Earth Resources Observatory and Science (EROS) Center) (public domain): http://eros.usgs.gov/#

Natural Earth (public domain): <http://www.naturalearthdata.com/>

**Response:** Thank you for the instructions. The satellite images were obtained from the database of AESA (Agência Nacional das Águas da Paraíba, Brazil). AESA uses the satellite images from Google Earth. We contacted the agency and explained about the Creative Commons Attribution License (CC BY 4.0), but it was explained to us that the satellite images used are not copyrighted and are in the public domain. The figure inserted next to the map is by the authors.

5. Please include captions for your Supporting Information files at the end of your manuscript, and update any in-text citations to match accordingly. Please see our Supporting Information guidelines for more information: http://journals.plos.org/plosone/s/supporting-information.

**Response:** Thank you for the instructions. The information was inserted in the text.

**Reviewer #1 done**

**Abstract**

The abstract needs some revisions for a clear understanding of the readers. Some specific comments are:

1) Line 20-21. Please remove this sentence as it seems out of context

**Response:** done

2) Line 25-27. Revise the lines please very vogue

**Response:** done

3) Line 27-28. Is it necessary to have hypothesis in abstract? Better to avoid such things in abstract

**Response:** Thanks for the suggestion. The hypothesis mentioned in the text of the abstract is related to the method. To build our phylogenetic tree we use a published tree. In evolution is called phylogenetic hypothesis.

4) Line 29. More like conclusion, if it’s added at the end of abstract as conclusion

**Response:** done

5) Conclusion missing

**Response:** Thank you for the suggestion. The text of the conclusion has been rewritten.

**Introduction**

6) The introduction is to the point but need language improvement in the sentence structure and also looks out for topographic mistakes. Some specific comments are

7) Line 54-55. Remove unnecessary words i.e. “in the first case”
**Response:** Done.

8) Line 64. By chance?? Is it necessary or important to mention.

**Response:** Yes. “by chance” makes reference to evolving Brownian motion.

9) Line 82-83. Please revise very vogue

**Response:** Thanks for the suggestion. The hypothesis mentioned in the text of the abstract is related to the method. To build our phylogenetic tree we use a published tree. In evolution is called phylogenetic hypothesis.

**Material and method**

10) Well and comprehensive written but need topographic correction. In addition, it lacks informations on the soil texture, electrical conductivity, and nutrient like organic matter and nitrogen which play critical role in the availability and accumulation of the heavy metals.
**Response:** Thanks for the suggestion. the information has been added.

11) Line 95. Revise please, very unnecessary verbs are used.

**Response:** Done.

12) Line 103. Past tense please, its manuscript methodology.

**Response:** Done.

13) Line 130-131. Please revise the sentence.

**Response:** Done.

14) Line 134-135. Very unnecessary sentence.

**Response:** Thanks for the suggestion. This information is important to detail the local ecological knowledge data.

15) Line 136-143. Please properly introduce your questionnaire and interview section i.e. classify the question section wise. Like respondents demography, social status, question about research etc.

**Response:** Thanks for the suggestion. A new subtopic has been inserted to present the interviews.

16) Line 166-167. Revise the sentence please

**Response:** Done.

**Results**

17) The portion is satisfactory, check for minor topographic corrections
**Response:** Done.

**Discussion**

The discussion is to the point, but need language improvement for better understanding of the readers. Some specific comments are.

**Response:** Thank you for your suggestion. The text has been revised to improve the language and a better understanding for the readers.

18) Page 16. As in our study, (44) observed that useful woody plants, phylogenetically close, located on the Amalfi Coast in Italy, were preferred for domestic practices due to tools production. (Please revise)

**Response:** Thanks for the suggestion. The quote would be to compare the results. Since it did not allow for contextualization we decided to remove it.

19) Page 17. Our results confirmed our prediction. The traits shared between the species mentioned for firewood and fences did not show a phylogenetic signal. Apparently this suggests that people tend to know less phylogenetically related species for these uses. Everything in the dry forest is useful in a situation of scarcity of species suitable for firewood. The literature shows that these uses demand larger amounts of plant biomass than categories that require less biomass, such as plants used in the production of technological artifacts (8,45). This may explain the reasons why there was no phylogenetic clustering. (Please revise, very poor writing).

**Response:** Thanks for the suggestion. Done.

20) Page 17. Same issue with the next paragraph.

**Response:** Done.

21) Page 18. Unlike studies that addressed utilitarian redundancy (Albuquerque & Oliveira 2006).

Correct the citation please.

**Response:** Done.

**Conclusion**

The conclusion needs to be refined and provide recommendations for further studies. In addition try to reduce the bulk of result repetition in conclusion.

**Response:** Thanks for the suggestion. Done.

**Note:** In addition to the reviewers' suggestions, we have inserted some excerpts, which are highlighted, in the text. In addition, we have changed the position of the topic.

Page 3 (Line 54-56)

Page 4 (Line 84)

Page 6 (Line 129-140)

Page 9 (Line 207)

Page 12 (Table 1)

Page 22 (discussion)

Page 23 (conclusions)

Page 25- 32 (references)

--------------------------------------------------------

Kamila Marques Pedrosa

State University of Paraíba, Department of Biology, Campina Grande, Paraíba, Brazil.
