# Supplementary material for: Plant parentage influences the type of timber use by traditional peoples of the Brazilian Caatinga
Source: PLoS One. 2023 Oct 17;18(10):e0286434. doi: 10.1371/journal.pone.0286434 (PMC10581497; doi:10.1371/journal.pone.0286434)
Supplement: Attachment — Submitted filename: Review 2.docx [file pone.0286434.s003.docx]

Abstract

Comments 1. Line 25-27 previously and now 23-25: We estimated the redundancy values for the mentioned species, and we compiled data from the literature on the wood density values of the cited species through a literature search.

Revise like… We estimated the redundancy values of the species and compiled data from the literature on the wood density values of the cited species.

Line 30-32: Not concluded properly

In addition, the language need thoroughly editing for improvement of the text
