# Supplementary material for: Plant parentage influences the type of timber use by traditional peoples of the Brazilian Caatinga
Source: PLoS One. 2023 Oct 17;18(10):e0286434. doi: 10.1371/journal.pone.0286434 (PMC10581497; doi:10.1371/journal.pone.0286434)
Supplement: Attachment — Submitted filename: Response Letters.docx [file pone.0286434.s004.docx]

To:  Dr. Tzen-Yuh Chiang

Academic Editor of PLOS ONE

Thank you for potentially accepting our manuscript entitled **“Plant parentage influences the type of timber use by traditional peoples of the Brazilian Caatinga”** for publication in PLOS ONE.

We would like to acknowledge the excellent suggestions from both reviewers for improving the revised version of our manuscript.

The suggested edits and comments made by the reviewers are shown below.

**Reviewer #2 done**

Abstract

Comments 1. Line 25-27 previously and now 23-25: We estimated the redundancy values for the mentioned species, and we compiled data from the literature on the wood density values of the cited species through a literature search.

Revise like… We estimated the redundancy values of the species and compiled data from the literature on the wood density values of the cited species.

**Response:** Thank you for the suggestion. Done.

Line 30-32: Not concluded properly

In addition, the language need thoroughly editing for improvement of the text

**Response:** Thank you for the suggestion. Done.
