# Supplementary material for: Plant parentage influences the type of timber use by traditional peoples of the Brazilian Caatinga
Source: PLoS One. 2023 Oct 17;18(10):e0286434. doi: 10.1371/journal.pone.0286434 (PMC10581497; doi:10.1371/journal.pone.0286434)
Supplement: Attachment — Submitted filename: Editor.docx [file pone.0286434.s005.docx]

Dear editor

The authors has substantially revises the manuscript (Plant parentage influences the type of timber use by traditional peoples of the Brazilian Caatinga") according to the suggestion and can be accept for publication.

Thank you

Dr. Rafi Ullah
